# Quest for the ideal assessment of electrical ventricular dyssynchrony in cardiac resynchronization therapy

**DOI:** 10.1016/j.jmccpl.2024.100061

**Published:** 2024-01-12

**Authors:** Uyên Châu Nguyên, Kevin Vernooy, Frits W. Prinzen

**Affiliations:** a Department of Physiology and Cardiology, the Netherlands; b Cardiovascular Research Institute Maastricht (CARIM), Maastricht University Medical Center (MUMC+), Maastricht, the Netherlands

**Keywords:** Cardiac resynchronization therapy, Heart failure, Electrocardiogram, Electro-anatomic mapping, Vectorcardiography

## Abstract

This paper reviews the literature on assessing electrical dyssynchrony for patient selection in cardiac resynchronization therapy (CRT). The guideline-recommended electrocardiographic (ECG) criteria for CRT are QRS duration and morphology, established through inclusion criteria in large CRT trials. However, both QRS duration and LBBB morphology have their shortcomings. Over the past decade, various alternative measures of ventricular dyssynchrony have been proposed, ranging from simple options such as vectorcardiography (VCG), ultra-high frequency ECG, and electrical dyssynchrony mapping to more advanced techniques such as ECG imaging electro-anatomic mapping. Despite promising results, none of these methods have yet been widely adopted in daily clinical practice. The VCG is a relatively cost-effective option for potential clinical implementation, as it can be reconstructed from the standard 12‑lead ECG.

With the emergence of conduction system pacing, in addition to predicting the outcome of conventional biventricular CRT, the assessment of electrical dyssynchrony holds promise for defining and optimizing the type of resynchronization strategy. Additionally, artificial intelligence has the potential to reveal unknown features for CRT outcomes, and computer models can provide deeper insights into the underlying mechanisms of these features.

## Highlights

• Methods for dyssynchrony evaluation ranges from simple options such as vectorcardiography to more advanced techniques such as ECG imaging.
• None of these dyssynchrony methods have yet been widely adopted in daily clinical practice.
• The VCG is a cost-effective option for clinical implementation, as it can be reconstructed from the standard ECG.
• Electrical dyssynchrony assesment can define the resynchronization approach, such as conventional biventricular or conduction system pacing.

## Abbreviations

**Table:** 

AHA	American Heart Association
ADV	activation delay vector
AI	artificial intelligence
CRT	cardiac resynchronization therapy
CSP	conduction system pacing
ECG	electrocardiogram
ECGI	electrocardiographic imaging
EDM	electrical dyssynchrony mapping
EAM	electro-anatomic mapping
ESC	European Society of Cardiology
HF	heart failure
HFrEF	heart failure with reduced ejection fraction
IVCD	intraventricular conduction delay
LBBB	left bundle branch block
LV	left ventricle
RBBB	right bundle branch block
RCT	randomized controlled clinical trials
SDAT	standard deviation of activation times
VCG	vectorcardiography
VEU	ventricular electrical uncoupling

### Study acronyms

**Table:** 

COMPANION	comparison of medical therapy, pacing and defibrillation in chronic HF;
Echo-CRT	echocardiography guided CRT;
LESSER-EARTH	evaluation of resynchronization therapy for HF
MADID-CRT	,multicentre automatic defibrillator implantation trial
MARC	markers and response to CRT;
MIRACLE	multi-center insync randomized clinical evaluation
MUSTIC	MULtisite stimulation cardiomyopathy;
NARROW CRT	narrow QRS ischemic patients treated with CRT
RAFT	resynchronization/defibrillation for ambulatory heart failure trial
RethinQ	resynchronization therapy in normal QRS;
REVERSE	reverse remodeling in systolic left ventricular dysfunction
SMART-AV	the SmartDelay determined AV optimization: a comparison to other AV delay methods used in CRT

## 1 Introduction

Cardiac resynchronization therapy (CRT) has significant impact on the trajectory of patients with dyssynchronous heart failure (HF) and is associated with left ventricular (LV) reverse remodeling and improved clinical outcome [1]. Identifying electrical dyssynchrony is important for patient selection in CRT. To define electrical dyssynchrony, it is essential to understand its meaning in the context of ventricular conduction disturbances. Invasive electrophysiological evaluation has revealed that a typical left bundle branch block (LBBB), which represents the ideal substrate for CRT, is characterized by uniform right-to-left activation with delayed ventricular conduction due to an increased duration of transseptal activation and slow LV myocardial activation [[2], [3], [4]]. This paper comprehensively reviews conventional and novel modalities for assessing electrical dyssynchrony, encompassing non-invasive techniques similar to the ECG to sophisticated mapping procedures. Additionally, relevant factors beyond electrical dyssynchrony assessment for disease modification after CRT, including mechanical dyssynchrony and the emergence of conduction system pacing (CSP), computational modeling, and artificial intelligence (AI), will be briefly discussed.

## 2 Electrocardiography

QRS duration and QRS morphology from the 12‑lead ECG remain the cornerstone in selecting patients for CRT. In the current European Society of Cardiology (ESC) guidelines [2], heart failure (HF) with reduced ejection fraction (HFrEF) patients under optimal medical therapy are eligible for CRT when they have a QRS duration of 130 ms or longer. Patients with a LBBB have a class or I or IIA indication for CRT, while patients with a non-LBBB have a class IIA or IIB indication.

Patients with HFrEF often present with intraventricular conduction delays, with a prevalence of wide QRS complexes in 25–50 % [2]. A prolonged QRS duration was originally interpreted as a surrogate for mechanical dyssynchrony and therefore used as an sole inclusion criteria for most randomized controlled clinical trials (RCT) that demonstrated the benefit of CRT including the MULtisite Stimulation Cardiomyopathy (MUSTIC) study [3], Multi-center Insync Randomized Clinical Evaluation (MIRACLE) study [4], Comparison of Medical therapy, Pacing and Defibrillation in Chronic HF (COMPANION) study [5], Reverse Remodeling in Systolic Left Ventricular Dysfunction (REVERSE) study [6], Multicentre Automatic Defibrillator Implantation Trial (MADID-CRT) [1], and Resynchronization/defibrillation for Ambulatory heart Failure Trial (RAFT) trial [7]. The minimal QRS duration used as an inclusion criterion varied between 120 and 200 ms. Patients with a more prolonged QRS duration demonstrated a more favorable outcome [8].

CRT trials that included patients with a narrow QRS (<120 ms) but with mechanical dyssynchrony on echocardiography including Resynchronization Therapy in Normal QRS (RethinQ) study [9], Narrow QRS Ischemic Patients Treated with CRT (NARROW CRT) study [10], Echocardiography guided CRT (Echo-CRT) study [11], and Evaluation of Resynchronization Therapy for HF (LESSER-EARTH) study [12], showed no or minor benefit to CRT. The latter two trials were even prematurely terminated for futility.

Based on the aforementioned findings, it can be inferred that CRT is most effective when a sufficient degree of conduction delay is present. QRS duration is generally used in forecasting disease modification after CRT but is still a crude measure of electrical dyssynchrony and does not provide any information about the underlying electrical mechanism. For this reason, more attention has shifted toward QRS morphology.

Post hoc analyses in the RCTs (MIRACLE, COMPANION, CARE-HF, RAFT, REVERSE, MADID-CRT) demonstrated that patients with a LBBB morphology benefit the most from CRT, while patients with a nonspecific intraventricular conduction delay (IVCD) or right bundle branch block (RBBB) only have little to no benefit [1,3,4,7,[13], [14], [15]].

Differences in LBBB definitions however exist (Table 1) [[1], [2], [3],^7^] and interpretation can be prone to subjectivity. In a study by van Stipdonk et al. [16] four device cardiologists evaluated 100 ECGs for the presence of LBBB based on clinical judgement. There was a relative weak (k = 0.35) agreement in LBBB identifications. The definition of LBBB has significant impact on clinical outcome and reverse remodeling in CRT recipients (Fig. 2A). While the ESC 2013 definition for LBBB is associated with the best clinical outcome compared to the Strauss, AHA and ESC 2021 definition [[17], [18], [19]]. LBBB definition introduced in ESC 2021 results in a significantly reduced proportion of patients meeting the criteria for baseline LBBB compared to the ESC 2013 definition. Interestingly, in a retrospective analysis of over 1200 patients, this change does not result in a more effective differentiation of CRT responders, nor does it establish a stronger association with clinical outcomes following CRT [17].

**Table 1:** Left bundle branch block definitions

Reference	Definition
ESC 2021	• QRS duration≥120 ms. • QS or rS in lead V1. • Broad (frequently notched or slurred) R waves in leads I, aVL, V5 or V6. • Absent Q waves in leads V5 or V6.
ESC 2021	• QRS > _120 ms. • Notches or slurring in the middle third of QRS in at least two of the following leads: V1, V2, V5, V6, I, and aVL - with a prolongation at the delayed peak in R in V5-V6 to longer than 60 ms. • Generally, the ST segment is slightly opposed to the QRS polarity, and particularly when it is at least 140 ms and is rapidly followed by an asymmetrical T wave also of opposed polarity. • Horizontal plane: QS or rS in V1 with small ‘r’ with ST slightly elevated and positive asymmetrical T wave and unique R wave in V6 with negative asymmetric T wave. When the QRS is <140 ms, the T wave in V6 may be positive. • Frontal plane: exclusive R wave in I and aVL often with a negative asymmetrical T wave, slight ST depression, and usually QS in aVR with positive T wave. • The QRS axis is variable.
AHA	• QRS ≥120 ms. • Notch-, slurred R in I, aVL, V5 and V6. • Occasional RS pattern in V5–6. • Absent q in I, V5–V6 and aVL. • R peak time > 60 ms in V5 and V6. • Normal R peak time in V1–V3. • No negative concordance. • Usually discordant ST-T segments.
Strauss	• QRS ≥130 ms in women, ≥140 ms in men. • QS or rS in V1 and V2. • Mid QRS notching/slurring in ≥2 congruent leads V1, V2, V5, V6, I or aVL.

Different definitions for LBBB according to the ESC 2013 guidelines [107], ESC 2021 guidelines [108], AHA [109] and Strauss criteria [19].

In a computational study the influence of heart-torso geometry on LBBB morphological criteria and QRS duration was investigated by creating five electrophysiological models of the heart based on real-time HFrEF patients with wide QRS rhythms [20]. Virtual geometrical modifications were subsequently induced in the models (translation and rotation of the heart, translation of the precordial electrodes), a source of variation that can only be induced in silico. Morphological characteristics on the ECG, for instance notching in the lateral leads were severely affected by these geometrical alterations and can influence the diagnosis of LBBB (Fig. 2B) [20].

In addition to QRS duration and morphology, other promising ECG characteristics, such as QRS fractionation [21], indications of residual left bundle branch conduction, the presence of S-waves in V6, and intrinsicoid deflection, have been proposed and requires further investigation in large clinical cohorts [22,23].

Taken collectively, the primary conduction disorder amenable to CRT is characterized by both prolonged QRS duration and LBBB morphology. While QRS morphology provides more detailed information about the underlying electrical substrate compared to QRS duration, the commonly used LBBB criterion can be susceptible to variations in definition, the subjective nature of ECG interpretation, and heart-torso geometry.

## 3 Vectorcardiography

**Fig. 1:**
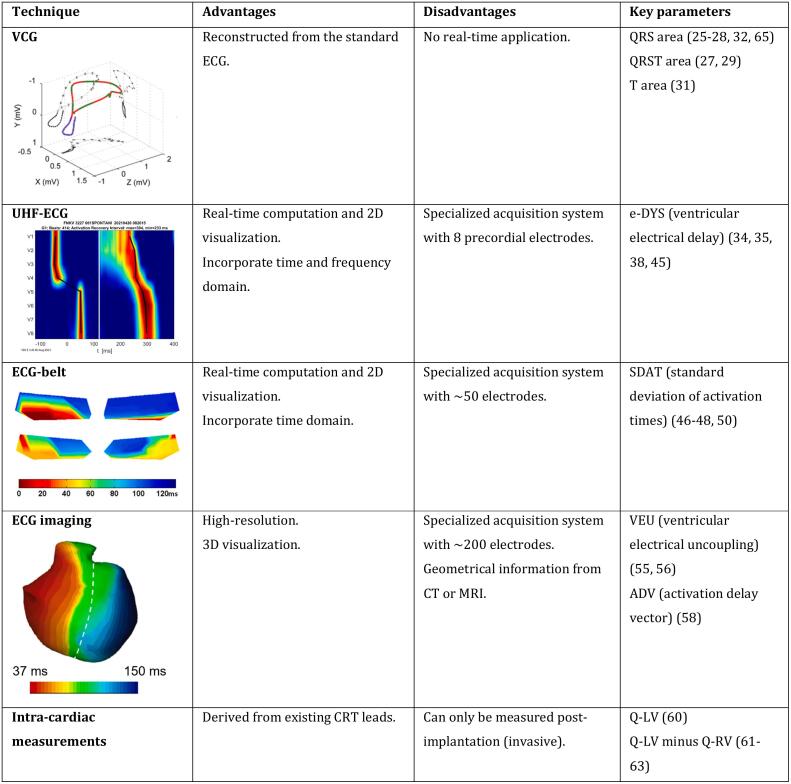
An overview of the most common methods used to evaluate electrical dyssynchrony between the standard ECG and invasive contact mapping, including their advantages, disadvantages, and key parameters. **Abbreviations:** ADV = activation delay vector, CRI = cardiac resynchronization index, EDM = electrical dyssynchrony mapping, LBBB = left bundle branch block, QRS duration = QRS duration, SDAT = standard deviation of all activation times, VEU = ventricular electrical uncoupling.

Over the past decade, multiple novel methods have been reported for the assessment of electrical dyssynchrony for patient selection in CRT (Fig. 1). A method that is close to the standard 12‑lead ECG is the three-dimensional (3D) vectorcardiogram (VCG). The VCG actually is an old method that recently resurged in popularity. It measures the electrical activity of the heart as a vector loop, consisting of momentary magnitudes and directions in 3D space for each time point in the heart cycle. The VCG can be derived from a true Frank VCG system (employing 7 recording electrodes) or reconstructed from the standard 12‑lead ECG with a mathematical transformation matrix [24]. The 3D area of the VCG QRS- (QRS-area) and T-wave loop (T-area) is presumed to reflect unopposed electrical forces during ventricular depolarization and repolarization, respectively. QRS-area from the reconstructed VCG is currently the most reported VCG parameter in identifying patient who will benefit from CRT.

**Fig. 2:**
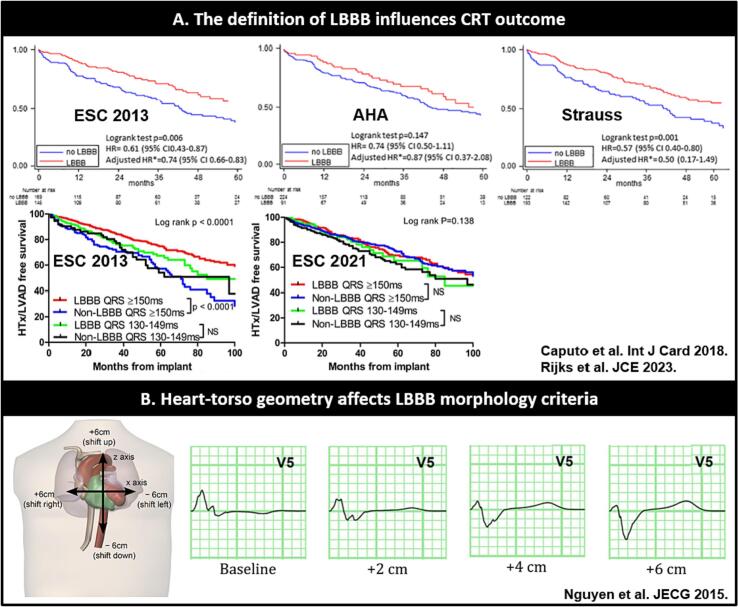
**A.** Combined survival estimates free of HFrEF hospitalization or all-cause mortality in 316 CRT recipients for three different LBBB definitions adapted from Caputo and Rijks et al. [17,18] Note that the definition of LBBB can impact CRT outcome. **B.** Computer simulation demonstrating how heart-torso geometry can influence the morphology of the ECG adapted from Nguyen et al. [20] In this representative simulation QRS notching in V5 (a morphological criterion for LBBB) disappears after shifting the heart upward.

**Fig. 3:**
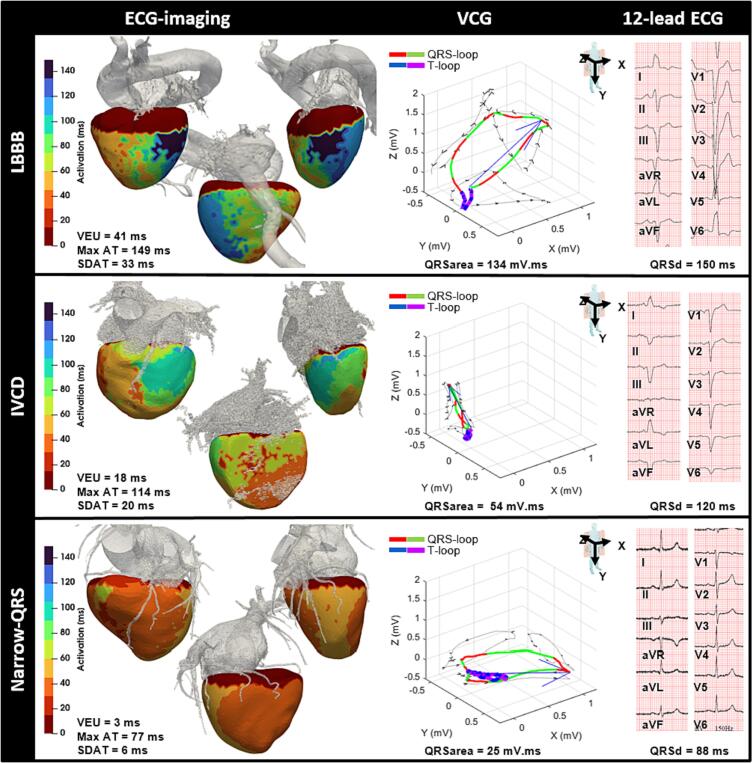
Preliminary data from our laboratory, illustrating dyssynchrony measures derived from ECGI, VCG, and the 12-lead ECG for a patient with LBBB (upper panel), IVCD (middle panel), and narrow-QRS (lower panel).

**Fig. 4:**
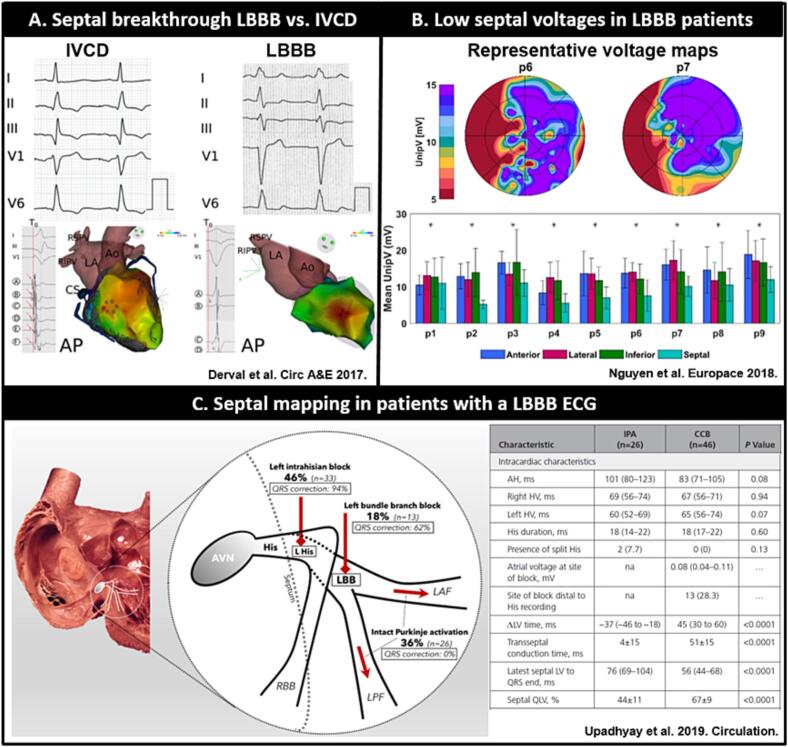
**A.** EAMs demonstrating septal breakthrough from an IVCD and LBBB patient adapted from Derval et al. [76] For the IVCD patient, the activation starts from multiple breakthroughs. Each of these breakthrough is associated with a sharp Purkinje potential. In patients with LBBB, activation starts later from a single breakthrough with no Purkinje potential. **B**. Representative voltage bullseye plots (up) and regional voltage amplitudes (down) from LV endocardial EAM in dyssynchronopathy patients without scar adapted from Nguyen et al. [74] Note that the voltage amplitudes are significantly lower in de septum. **C.** Sites of conduction block in patients with LBBB pattern with rate to response to corrective HBP (left) and intracardiac characteristics between patients with a complete conduction block (CCB) and intact His Purkinje activation (right) adapted from Upadhyay et al. [77] Among patients diagnosed with CCB, the blockage was frequently observed in the left-sided His fibers, and such location was identified as the most feasible site for HBP. However, in some cases, CCB was observed in areas beyond the His recording. Such sites were less amenable to HBP and were consistent with blockage in the distal branching bundle or proximal left bundle-branch. The remaining group of LBBB patients did not exhibit CCB.

Van Deursen et al. conducted VCG measurements in 81 CRT candidates using the conventional Frank orthogonal leads system and demonstrated that a QRS-area ≥ 98 μVs was a superior predictor of echocardiographic CRT outcome compared to QRS duration and LBBB morphology [25].

The association between QRS-area from the reconstructed VCG and clinical and echocardiographic outcomes was also confirmed in a second retrospective multicenter study with almost 1500 CRT recipients. A QRS area ≥ 109 μVs identified clinical and echocardiographic outcome more effectively than QRS morphology and QRS duration and was the only independent electrocardiographic determinant associated with the clinical outcome [26]. A large retrospective Danish study of 705 CRT recipients consistently demonstrated that a QRS area ≤ 95 μVs is associated with a decreased clinical outcome. This association holds true regardless of QRS duration and the presence of LBBB morphology, emphasizing the importance of QRS area as a valuable prognostic marker in CRT [27]. Moreover, in the MARC study (Markers and Response to CRT) QRS-area besides echocardiographic intraventricular mechanical delay and apical rocking was prospectively associated with LV reverse remodeling at six months in the multivariate model [28]. Sub-analyses of these studies indicate that QRS-area could be particularly of added value in identifying non-LBBB patients who do benefit from CRT. The sequential MARC-2 study (currently ongoing) will therefore investigate whether QRS-area could prospectively predict CRT outcome particularly in non-LBBB patients with prolonged QRS duration (NCT04120909).

Other VCG parameters, incorporating repolarization rather than activation, have also been proposed for predicting CRT outcomes. In the SmartDelay Determined AV Optimization: A comparison to other AV delay methods used in CRT (SMART-AV) trial, 234 CRT recipients were studied. Patients with a high sum of the absolute QRST integral (QRST-area) in the third tertile had 1.9 times greater odds of LV reverse remodeling than those with the lower two tertiles combined (OR 1.9, 95 % CI 1.1–3.5; *p* = 0.03) [29].

QRST-area was similarly associated with LV reverse remodeling in a British and Danish CRT cohort, although it was outperformed by QRS-area [27,30].

In a retrospective study of 335 CRT recipients conducted by Vegh and Engels et al., the patient subgroup with a T-wave area ≥ median and LBBB showed a significantly lower incidence of the primary clinical endpoint compared to patients with LBBB and a small T-wave area or non-LBBB patients with a small or large T-wave area (48 %, 57 %, and 51 %, respectively) [30,31]. In the MARC study, a significant association between T-area and LV reverse remodeling was present in the unadjusted model, but T-area was outperformed by QRS-area in the multivariate model [28]. These results suggest that the relationship between repolarization and CRT is not yet fully understood.

Taken together, there is increasing evidence that a large QRS-area is associated with beneficial clinical and echocardiographic outcomes after CRT and that it therefore has additional value in patient selection for CRT. It is not yet clear what QRS-area truly reflects in the heart is not fully understood. Therefore, investigating the relationship between QRS-area and specific local anatomical, tissue, and electrical characteristics could provide valuable mechanistic insights.

In one of the early VCG-CRT studies, QRS-area was reduced in patients with ischemic cardiomyopathy compared to those with a non-ischemic etiology, suggesting an association between low QRS-area and myocardial scar [25]. The association between QRS-area and myocardial scar from CMR was therefore investigated in 33 recipients of CRT. QRS-area inversely correlated with focal scar, but not with diffuse fibrosis on CMR. Additionally, patients with low focal scar burden and high QRS-area (≤66 μVs) benefited the most from CRT. These findings could indicate that scar tissue with higher density may lead to a decrease of total electrical forces during the cardiac cycle and concordantly a decrease of VCG amplitude. QRS-area in this sense could potentially reflect myocardial viability [30].

There has been limited research into the association between QRS-area and intra-cardiac electrophysiological characteristics. QRS-area has shown the potential to detect delayed activation of the LV lateral wall, which is considered the substrate remediable through CRT [32]. QRS-area > 69 μVs diagnosed delayed LVLW activation with a higher sum of sensitivity (87 %) and specificity (92 %) than any of the LBBB definitions. Delayed LV lateral wall activation was in this case defined as local activation time, measured by coronary venous EAM, exceeding >75 % of QRS duration on the surface ECG [33].

Taken collectively, data from large but mostly retrospective studies indicate that VCG QRS-area is a promising quantitative parameter that may be superior to conventional CRT selection criteria, like QRS duration and LBBB morphology. The mechanistic basis of QRS-area appears to lie in its ability to reflect low myocardial scar burden, and delayed LV activation. Ongoing prospective studies exploring the potential of QRS-area as a predictor for CRT outcome in a prospective setting, and further investigations into the mechanism of QRS-area are warranted.

## 4 Ultra-high frequency ECG

The ultra-high-frequency ECG (UHF-ECG) technique was first introduced by Jurak et al. in a proof of concept study in seventeen patients prior and post CRT implantation as a more refined method for assessing electrical dyssynchrony [34,35]. Standard ECG systems traditionally record within the bandpass range of 0.05–100 Hz or 0.50–150 Hz with six precordial leads and three limb leads. The UHF-ECG technique record ECG signals with high sampling rate and a band width of up to 1500 Hz. The ECG is analyzed in 16 frequency bands (width 100 Hz, step 50 Hz, range 150–1050 Hz) from eight precordial leads (V1-V8) [36].

To create a broad-band QRS complex the average of sixteen normalized median (over multiple beats) amplitude envelopes is computed and visualized as a color map for each lead. UHF-QRS duration at 50 % of its amplitude is used to compute the local depolarization time for each precordial lead. Ventricular electrical delay (e-DYS) is calculated as the maximum difference between the center of mass of UHF-QRS (local depolarization times) between leads V1-V8 [34]. In the initial UHF-ECG paper by Jurak et al. e-DYS >50 ms identified a reduction of 10 % or more in LVESV for all CRT recipients [34].

Plesinger et al. conducted retrospective validation of the UHF-ECG technique in a large patient cohort from the MADID-CRT trial [37]. An e-DYS of ≥31 ms was significantly associated with a higher risk of HF or death in the LBBB patients, whereas these associations were only borderline significant for IVCD patients and not significant for RBBB patients [38].

Since its validation in the MADIT-CRT population, UHF-ECG derived ventricular electrical delay has been utilized as a measurement of dyssynchrony in multiple clinical studies, [[39], [40], [41], [42], [43], [44], [45]] especially in exploring the ventricular activation patterns in different approaches of CSP.

UHF-ECG can be applied to clinical practice with minimal patient burden, but it necessitates a specialized system capable of capturing high-frequency potentials. Its primary advantage is the real-time evaluation and visualization of dyssynchrony, which can prove particularly beneficial during implantation and optimization of any forms of CRT (e.g. biventricular or CSP).

## 5 Body surface potential mapping

The ECG-belt system is a mid-level solution that bridges the gap between the traditional 12‑lead ECG and the comprehensive ECGI, utilizing ∼50 body surface electrodes. The standard deviation of all ECG-belt derived activation times (SDAT) has been proposed as a new metric for assessing electrical dyssynchrony, which presumably reflects electrical heterogeneity. In a study of 66 CRT recipients, patients with SDAT ≥35 ms had significant greater increase in LVEF and reduction in LVESV after CRT, unlike QRS duration or morphology [46]. Moreover, SDAT had much better accuracy (sensitivity 90 %, specificity 80 %) for identifying hemodynamically responsive sites compared with RV-LV delay (69 %, 85 %) or paced QRS reduction (52 %, 76 %) [47].

An RCT involving over 400 patients with primarily a class II indication for CRT investigated, the use of the ECG-belt to guide implantation and programming of CRT (intervention group) versus current standard of care (control group). Combining the control and ECG-belt arms for linear regression analyses showed that baseline SDAT was superior to QRS duration in predicting change in LV reverse remodeling, particularly in non-LBBB patients. Moreover, despite a significant CRT-induced reduction in SDAT in the intervention group compared to the control group, there was no significant difference in LV reverse remodeling between the two groups. Patients in the ECG Belt arm had a median decrease in SDAT of 46.8 % (IQR 27.8 %–67.3 %), while the median decrease in SDAT for control patients was 19.4 % (IQR −10.5 % to 41.1 %). This indicates that while baseline electrical dyssynchrony is an important factor for disease modification after CRT, improvement in SDAT is not directly related to improved LV function [48]. There are several possible reasons for these findings, including the likelihood that SDAT primarily indicates intraventricular (diffuse) dyssynchrony rather than interventricular dyssynchrony, the absence of significant baseline dyssynchrony that could be corrected, or the possibility that the structural heart disease in patients with a class II indication is too advanced for reverse remodeling to occur. Conversely, in a multi-center registry involving 1299 patients, a decrease in dyssynchrony as defined by delta VCG QRS-area was found to be a distinct predictor of CRT response, particularly in patients with a significant baseline QRS-area [49].

A derivative method from the ECG-belt was recently proposed by Banks et al. where signal data from 18 leads were converted in an electrical dyssynchrony map (EDM) that displayed the atrioventricular delay vs. de interventricular delay in 2D color-coded for a cardiac resynchronization index that was derived from the QRS area [50]. EDM was able to optimize CRT in 39 patients who initially exhibit suboptimal response, improving echocardiographic outcome [51].

To summarize, while it is widely accepted that baseline dyssynchrony can predict outcomes, the usefulness of post-implantation programming optimization guided by a reduction in dyssynchrony is subject to debate and may depend on the method used to evaluate dyssynchrony.

## 6 ECG imaging

ECG imaging (ECGI) is a more complex technology that reconstructs the electro-anatomic activation of the epicardium using approximately 200 body surface potential measurements and a personalized heart-torso geometry derived from CT or MRI [52]. Several commercial and non-commercial ECGI systems have been developed. In our laboratory, we use the “Maastricht ECGI system” which has been validated with contact mapping in anesthetized dogs [53]. Fig. 3 shows representative activation maps from two CRT recipients and a patient with a structurally normal heart obtained using the ECGI system from Maastricht.

Studies by the Bordeaux group showed that the overall agreement between the commercial CardioInsight ECGI system and traditional epicardial contact mapping was disappointing and particularly heterogeneous in regions with scar. On the other hand, better agreements were obtained in wider QRS and paced rhythms, indicating that ECGI has potential for use in patients with ventricular conduction delays [54]. Experiments from the same group with Langendorff-perfused pig hearts suspended in a torso tank additionally showed that ECGI can precisely identify electrical dyssynchrony, detect resynchronization caused by BiVP, and locate the site of latest activation with high accuracy [55].

In one of the earliest studies examining the effectiveness of ECGI in CRT, the parameter ventricular electrical uncoupling (VEU), indicating the difference between mean activation times of the LV and RV, was investigated. A VEU >50 ms demonstrated a stronger association with improved clinical composite outcomes compared to total LV and RV activation times, as well as QRS duration and morphology. VEU was 72 ± 16 ms in CRT responders, whereas it was 38 ± 23 ms (*p* = 0.001) in non-responders to CRT [56]. Computer simulations conducted more recently revealed that isolated interventricular dyssynchrony, as represented by VEU, plays a more significant role in driving acute hemodynamic response during intrinsic rhythm than total activation times of the RV and LV. Interestingly, changes in total RV and LV activation times during BiVP had minimal impact on acute hemodynamics [57].

The activation delay vector (ADV) is another measure of electrical dyssynchrony obtained from ECGI. It quantifies the amount and direction of activation delay in three dimensions. To calculate ADV, vectors are generated from all virtual epicardial nodes using the center of the heart, and these subvectors are multiplied by their corresponding activation time. The resulting subvectors are then summed to obtain the ADV, whose angle is calculated in both the frontal and transversal planes. Notably, the direction of dyssynchrony as indicated by ADV is similar for patients with LBBB and IVCD, although the ADV is larger in LBBB.

A baseline ADV >89 ms has been demonstrated as a superior predictor of clinical outcomes compared to both LBBB morphology and QRS duration. In predicting acute hemodynamic response, a baseline VEU of >61 ms and ADV of >95 ms were found to be superior to baseline QRS duration. However, the impact of varying LV pacing sites on acute hemodynamic response is only minor, with an observed increase of +3 ± 4 % in LV dP/dtmax compared to conventional basolateral LV pacing. [58].

Taken collectively, current data indicates that ECGI has the capability to identify the electrical substrate for CRT with greater accuracy than the 12‑lead ECG, which potentially enhances patient selection. Similar to the ECG-belt, ECGI has potential to outperform the 12‑lead ECG with patient identification through baseline dyssynchrony assessment, but its capacity to further enhance benefit within a patient by varying pacing sites is only minor. The practical application of ECGI for this purpose furthermore necessitates specialized imaging and post processing, making it more challenging for routine clinical use.

## 7 Electrophysiological evaluation

### 7.1 Q-LV and delayed LV activation

As previously mentioned, CRT is primarily employed to treat conduction disorders that are characterized by a prolonged QRS duration and exhibit an LBBB morphology on the surface ECG. It is not surprising that prolonged QRS duration could be indicative of delayed LV activation. The relationship between local LV electrical delay and CRT disease modification was evaluated in 71 CRT recipients by Singh et al. [59]. Electrical delay was defined as the interval from onset of the QRS on the surface ECG to the onset of the sensed EGM on the LV (Q-LV) and expressed as a percentage of baseline QRS duration. Q-LV was significantly longer in acute responders (responders 70 ± 24 %, non-responders 32 ± 12 %, *p* = 0.002) among patients with non-ischemic cardiomyopathy [59]. Gold et al. similarly demonstrated in 426 CRT recipients from the SMART-AV trial that Q-LV was associated with LV reverse remodeling and clinical outcome Patients in the highest quartile of QLV had a > 3 fold increase in their odds of LV reverse remodeling after correcting for QRS duration, bundle branch block type, and clinical characteristics by multivariate logistic regression analysis [60]. The latter group, and more recently also the Aarhus group, additionally showed that baseline interventricular delay, measured as the duration between the sensed RV and LV lead EGM was also associated with LV reverse remodeling and clinical outcome [[61], [62], [63]].

Mafi-Rad et al. performed coronary venous EAM in patients with LBBB and IVCD and showed that delayed LV activation (defined as Q-LV >75 % of QRS duration) could be identified in 24 out of 25 LBBB patients and 12 out of 23 IVCD patients [64,65]. In both the LBBB and IVCD patients, the latest activated region varied between basal-mid anterolateral-inferolateral walls. These findings consistently suggest that delayed LV activation is strongly associated with a LBBB activation pattern but this electrical substrate may also be present in half of the IVCD patients.

One must consider that delayed LV activation may coincide with myocardial scarring, suggesting that it may arise from damage to the functional myocardium rather than an impaired rapid conduction system, which is not the primary dyssynchrony substrate for CRT. In two studies DE-CMR was integrated with coronary venous EAM and ECGI, respectively, in a total of 34 consecutive CRT recipients. In approximately one-third of the patients had the latest activated region located within myocardial scar [66,67].

### 7.2 Contact mapping

Cardiac mapping to identify key patterns of a LBBB activation in humans has a rich history spanning many years. Vassallo et al. conducted one of the first endocardial catheter mapping studies of LBBB. Their findings showed that LBBB is caused by right-to-left transseptal activation and is associated with significant heterogeneity in LV endocardial activation [68]. Narula measured prolonged HV times in LBBB patients regardless of etiology and found that stimulating the His bundle abolished the LBBB pattern. He therefore suggested that a LBBB pattern may result from a focal area of altered refractoriness within the His bundle [69]. More detailed RV and LV endocardial 3D EAM studies by Rodriguez et al. identified two types of septal activation in LBBB patients with HFrEF: through slow conduction through the left bundle or via right-to-left transseptal activation. The latest activation was present in the LV basal-posterior or –posterolateral wall [70]. Auricchio et al. performed RV and LV endocardial EAM in HFrEF patients with LBBB. In 23 out of 24 patients a u-shaped conduction pattern on the LV activation was reported by a transmural functional line of block located between the LV septum and the lateral wall. The morphology of the EGM in these regions of block appeared rather fractionated even though the majority of the patients had idiopathic cardiomyopathy and lacked the presence of myocardial scar. This fractionation also disappeared when the wavefront of activation was changed by pacing and (near) normal bipolar and unipolar EGMs were present in the regions surrounding the line of block [71]. It is worth noting that a considerable range of transseptal conduction times were observed among patients referred for CRT. Although some patients displayed near-simultaneous activation at both the RV and LV (transseptal time close to 0), the majority of patients with LBBB exhibited transseptal times >30 ms. Higher transseptal times were associated with more prolonged QRS duration but also a mid- to apical-septal LV breakthrough site, which may indicate a myocardium mediated activation sequence from the RV to LV [72]. Strik et al. concordantly performed detailed LV endocardial contact and non-contact mapping and epicardial contact mapping in canine models with acute LBBB and chronic LBBB with tachypacing-induced HFrEF. Transseptal times were significantly longer in the LBBB-HF models compared to the acute LBBB models [73]. The mechanism underlying the slow conduction across the septum can be partly explained by the conduction direction being perpendicular to the fibers. The septum conceivably exhibits distinct structural anomalies that give rise to anisotropic conduction, particularly in the aftermath of remodeling attributable to dyssynchronopathy.

Notably, a consistent reduction in unipolar voltage amplitudes was observed in the septum during a LBBB activation sequence in both HFrEF patients and computer models without myocardial scar (Fig. 4B) [74]. These low septal voltage amplitudes were affected by changing the wavefront of activation through ventricular pacing. This observation may support the concept of anisotropic conduction properties of the septum and could potentially account for the moderate to poor association between unipolar voltage amplitudes and scar in patients with dyssynchronopathy and prolonged transseptal conduction oberved by Auricchio et al. [71,75].

A study conducted by Derval et al. compared EAM in HFrEF patients with LBBB, IVCD, and narrow-QRS ECG. In their data, LBBB patients showed a delayed onset of activation in the LV, which originated from a single breakthrough site in the mid-septal area and was not preceded by Purkinje potentials (Fig. 4A). The activation of the entire LV was dependent on the properties of myocardial conduction, and the spread of activation was homogeneous without significant areas of slow conduction, consistently ending again at the LV basal lateral wall. Narrow-QRS and IVCD patients on the contrary demonstrated rapid and multifocal onset of LV activation mediated by Purkinje fibers. The late components of LV activation in these patients were mostly due to slow conduction in areas of myocardial scar. Interestingly, areas of late activation were not always directly associated with regional scar but were occasionally the result of upstream slowing of the activation wavefront by remote scar [76].

More recently Upadhyay et al. performed LV septal mapping in 76 patients with a surface LBBB pattern and 16 controls (Fig. 4C). Among patients with a LBBB pattern, heterogeneous septal conduction was observed. A complete conduction block within the proximal left conduction system was present in 64 % of patients, while an intact Purkinje activation was observed in the remaining 36 % of patients. His Bundle pacing (HBP) corrected wide QRS in 85 % of patients with a complete conduction block, and in none of the patients with an intact Purkinje activation. Patients with a complete conduction block showed more presence of QRS notching and more often met the Strauss criteria on the surface ECG and had a higher transseptal conduction time and higher septal QLV during intra-cardiac mapping compared to those with an intact Purkinje system [77].

Another recent study by Maffessanti et al. studied electro-mechanical mapping in 62 CRT recipients, with a predominantly LBBB surface ECG. They found that echocardiographic reverse remodeling after CRT was linked to a prolonged transseptal time, short total LV activation, the lack of myocardial scarring on DE-CMR, and a high systolic stretch index [78]. The observation of the link between short LV activation time and reverse remodeling may be surprising, but can be explained by good conduction properties of the normal LV myocardium.

Taken collectively, electrophysiological studies demonstrate a correlation between LV electrical delay and interventricular delay with LV reverse remodeling and clinical outcomes following CRT. The “true” activation pattern of a proximal LBBB, and thus presumably ideal substrate for CRT, may be characterized by a single breakthrough in the septum, extended transseptal conduction, and a uniform LV activation pattern through working myocardium, with the latest activation occurring in the basal-inferolateral wall of the LV. However, contact mapping has shown that patients with an LBBB pattern on the surface ECG may exhibit varying degrees of conduction disturbance and sites of latest activation.

## 8 Beyond electrical dyssynchrony

While the primary focus of this review was to provide an overview of electrical dyssynchrony methods in CRT, we would also like to briefly discuss other relevant factors in disease modification after CRT.

### 8.1 Myocardial scar

The presence, especially pacing, in myocardial scar has been linked to reduced outcomes in CRT. When pacing within myocardial scar, slow conduction can interfere with resynchronization, leading to reduced efficacy from an electrophysiological perspective. In a large clinical study involving 559 CRT recipients, pacing within cardiac magnetic resonance (CMR) defined scar was associated with a higher incidence of cardiovascular death, sudden cardiac death, and lower LV reverse remodeling compared to pacing outside of scar [12].

### 8.2 Mechanical dyssynchrony

Echocardiographic measures of mechanical dyssynchrony have undergone extensive investigation, though current guidelines do not recommend their use for patient selection for CRT [79].

The EchoCRT trial showed that CRT in patients with peak-to-peak dyssynchrony assessed by tissue Doppler or radial strain and a narrow QRS width (<130 ms) was not beneficial and potentially harmful. [80].

The systolic stretch index, as determined by strain imaging, combines the systolic prestretch of the lateral wall resulting from early septal contraction and the septal rebound stretch caused by late lateral wall contraction. In 442 patients enrolled in the Adaptive CRT trial, a high systolic stretch index (>group median of 3.1 %) was significantly associated with freedom of HF hospitalization or death [81].

Moreover, in the MARC study echocardiographic intraventricular mechanical delay and apical rocking was prospectively associated with LV reverse remodeling in the multivariate model [28].

Layec et al. concordantly demonstrated that indices of mechanical dyssynchrony including septal flash, apical rocking, septal deformation patterns, and global wasted work determined with speckle tracking strain echocardiography, enhanced the prediction of clinical outcomes to CRT in 551 patients [82]. Bivona additionally illustrated that a poor prognosis after CRT in a cohort of 102 patients was linked not only to lack of delayed mechanical activation (<34 ms) but also to neurohormonal factors and kidney failure [83].

### 8.3 Time and frequency domain

The focus on assessing electrical dyssynchrony for CRT has primarily revolved around the duration and morphology characteristics of a single-beat ECG. Several studies have suggested incorporating a broader frequency domain [34], beat-to-beat dynamics [22], and velocity components (first derivative) [84] in dyssynchrony assessment.

Zhan et al. recently calculated R-wave singularity (discontinuity of R-waves) from Holter ECG recordings. They demonstrated that R-wave singularity was significantly less negative in 93 patients with myocardial infarction and electrical dyssynchrony compared to 202 normal subjects, suggesting a more heterogeneous depolarization of the myocardium [22].

Fernández et al. proposed incorporating the first derivative (velocity) integrals of the VCG in dyssynchrony analyses, introducing a “ventricular dyssynchrony index.” This index is defined as the disparity between the voltage and speed time integrals of an individual observation and the linear fit of these variables derived from a healthy population. Despite its innovativeness, the clinical potential of this ventricular dyssynchrony index still needs evaluation in clinical cohorts [84].

### 8.4 Conduction system pacing

CSP consists of different novel pacing techniques, more specific his-bundle pacing (HBP) and left bundle branch area pacing (LBBAP). Both techniques have been introduced as a more physiologic alternative to anti-bradycardia pacing and biventricular (BivP) CRT. [42,43]

The most physiological form of pacing is theoretically provided by HBP, as there is complete recruitment of the conduction system to both ventricles [44]. In relatively small RCTs, patients who underwent HBP showed superior electrical resynchronization and a tendency toward greater LV reverse remodeling [85], along with more significant clinical and physical improvements compared to BiVP. However, this advantage was accompanied by a higher incidence of capture loss or rising thresholds [86].

LBBAP, comprising of LV septal pacing (LVSP) and left bundle branch pacing (LBBP), may create inferior electrical synchronization by inducing RV dyssynchrony, compared to HBP. However, LBBAP is considered a more practical alternative due to its ease of implementation and is associated with greater electrical stability [87]. LBBAP in patients with dyssynchronous HF has been associated in small RCTs and larger observational registries with stable pacing thresholds, improved echocardiographic [88] and clinical outcome [[89], [90], [91]], and lower incidence of ventricular arrhythmias [90] compared to BivP.

While CSP-CRT appears promising, the number of randomized trials and included patients remains limited. Long-term data on clinical outcomes and complications are still pending. Consequently, conventional BiVP remains the guideline-recommended treatment for dyssynchronous HF. However, CSP may be a viable alternative to conventional BiVP in situations where traditional CRT implantation is unsuccessful.

Santoro et al. presented an algorithm to guide the choice between conventional CSP-CRT or BiVP-CRT. They suggested CSP when the LV lead could not be placed in a lateral or inferolateral vein or when RV sense - LV sense ≥100 ms, RV pace – LV sense ≥120 ms, or LV pace – RV sense ≥110 ms. In the cohort guided by the algorithm, approximately 25 % of patients met the criteria for CSP-CRT. Additionally, applying this treatment algorithm for CRT led to enhanced clinical outcomes compared to a historical CRT cohort [92].

Non-invasive simultaneous electrical dyssynchrony methods can play an important role during in LV guidance during CSP.

### 8.5 Computer modeling

Realistic electrophysiological models of the heart can provide detailed information on the underlying mechanism of dyssynchrony and its correction by CRT. Some models are highly intricate, necessitating substantial computational resources. Conversely, simpler models can be solved more efficiently. Modeling multiscale electromechanical function, spanning from the cellular to organ level, typically involves two coupled parts. These simulate the electrical aspect (monodomain/bidomain based on cellular ionic models) and the mechanical aspect (continuum mechanics based on cellular myofilament models) of the heart, respectively. The intracellular calcium released during electrical activation serves as the coupling mechanism between the electrical and mechanical components of the model [93,94].

Potse et al. developed a high-end reaction-diffusion model using a bidomain framework that incorporated patient-specific anatomies, that was utilized to build patient tailored dyssynchrony models [20]. By solving the bidomain equation, the model enabled the computation of surface ECG leads and intra-cardiac electrograms from simulated action potentials. A comprehensive parameter tuning process was conducted to accurately simulate the measured EAM and surface ECG signals [95].

A simpler rule-based electrophysiological model employs predefined rules to define the electrophysiological parameters. The model uses mathematical equations to describe electrical activity of the heart and can be customized to simulate specific cardiac pathologies. Lee et al. examined the role of rule-based methods in defining the electrical properties of the heart in 18 dyssynchrony patients. They identified fast endocardial conduction as the most crucial factor for achieving physiologically plausible parameters [96]. An even more computational efficient model is the Eikonal model. The Eikonal model is a mathematical model that computes the electrical activation time in the heart based on the propagation of a wavefront. It assumes that the heart's conduction properties can be represented by a scalar field, and the wavefront propagates through this field according to the Eikonal equation. Impressively, through an iterative optimization process, Pezzuto et al. was able to reconstruct the 3D ventricular EAM by utilizing a patient-specific Eikonal model. This was accomplished by optimizing conduction velocity and sites of earliest activation to match the simulated ECG to the recorded 12‑lead ECG and CMR data [97].

Khamzin et al. similarly reconstructed 3D ventricular activation maps tailored to heart:torso geometry using MRI and CT, along with standard ECG data from 57 patients. The utilization of a combination of clinical and model-driven data could predict a > 10 % increase in LVEF one year post-implantation with an AUC of 0.82. The distance from the LV pacing site to the post-infarction zone and ventricular activation characteristics under BiVp pacing were identified as the most relevant model-driven features for CRT response classification [98]. In cases where biophysical processes are not yet fully understood, mechanistic models can be augmented with AI, driven by population-based data [94,99].

### 8.6 Artificial intelligence

Artificial Intelligence (AI) models applied to standard ECGs have outperformed cardiologists in detecting concealed cardiovascular pathology [[100], [101], [102], [103]]. Despite these encouraging advances, the implementation of AI in clinical settings is impeded by the absence of reliable methods to explain the algorithms to healthcare practitioners. Van de Leur et al. introduced an innovative pipeline employing variational auto-encoders to comprehend the fundamental factors from the median beat ECG morphology, referred to as FactorECG. FactorECG was trained on a database of 1.1 million ECGs, and the variational auto-encoder extracted 21 generative ECG features that are commonly associated with physiological process, which were subsequently integrated into conventional prediction models. The uniqueness of this pipeline is that all incorporated ECG factors were made explainable by interactively visualizing these morphological changes in both the model as well as for the individual patient ECG [104].

AI models have not been extensively used in the field of CRT. Wouters et al. from the same Utrecht group converted 1306 ECGs from CRT patients' prior implantation into their respective FactorECG. The combined clinical endpoint of death, LV assist device or heart transplantation was predicted by FactorECG with c-statistic of 0.69 outperforming guideline ESC criteria (c-statistic 0.57) and even QRS area (c-statistic 0.61). Additional predictive ECG features associated with poor outcome identified were inferolateral T-wave inversion, smaller right precordial S- and T wave amplitudes, ventricular rate, increased PR interval and P-wave duration [105]. These findings suggest that besides focusing on interventricular dyssynchrony, atrioventricular and atrial dyssynchrony and repolarization play an important role in CRT outcome prediction as well. In a smaller study, Howell et al. applied 8 different machine learning models CRT patients from the SMART-AV trial (∼600 training and ∼ 150 testing). The best model (adaptive lasso) was able to predict a composite endpoint of LV reverse remodeling, survival and HFrEF hospitalization at 6 months post CRT with an accuracy of 70 % [106].

AI has the potential to assist clinicians in tasks that are beyond their own capabilities and uncover mechanisms that can only be revealed through a data-driven approach. In turn, clinicians can train the AI models. While the use of AI in clinical practice is not yet widespread, it has promising potential in the area of CRT from identifying patients to predicting disease outcomes based on a chosen therapy.

## 9 Conclusion

The currently recommended ECG criteria for CRT include a prolonged QRS duration and LBBB morphology. Invasive electrophysiological studies revealed that a LBBB pattern on the surface ECG may exhibit varying degrees of conduction disturbance, transseptal times and breakthrough and latest activation sites. There have been numerous proposals for alternative measures of ventricular dyssynchrony over the past two decades, many of which have demonstrated superior diagnostic performance compared to traditional QRS duration and morphology. Proposed alternatives including VCG, EDM, UHF-ECG, ECG-belt and ECGI have shown superior predictive performance compared to traditional QRS duration and morphology in forecasting CRT outcomes. VCG is the most accessible option since it can be reconstructed from the standard 12‑lead ECG and does not necessitate the acquisition of new hardware.

In addition to predicting the outcome of conventional CRT implantation, simultaneous assessment of electrical dyssynchrony holds promise in guiding CSP procedures. This innovative approach to resynchronization offers a broad spectrum of lead position options. The emergence of AI has opened up the possibility of revealing other relevant mechanisms for CRT outcome. Moreover, patient-specific computer models, ideally based on mechanistic models augmented with AI driven by population-based data, hold promise for providing more insights into the relationship between these uncovered features and CRT outcome.

## Funding

U.C. Nguyen was supported by a personal grant from the Dutch Heart Foundation (2021T016).

## Compliance with the ethical standards

This manuscript provides a literature overview and does not contain any direct studies involving human participants. Ethical approval and informed consent are therefore not applicable.

## CRediT authorship contribution statement

**Uyên Châu Nguyên:** Conceptualization, Visualization, Writing – original draft, Writing – review & editing. **Kevin Vernooy:** Conceptualization, Resources, Supervision, Writing – review & editing. **Frits W. Prinzen:** Conceptualization, Resources, Supervision, Writing – review & editing.

## Declaration of competing interest

F.W. Prinzen reports research grants from Medtronic, Abbott, MicroPort CRM and Biotronik. K. Vernooy declares consultancy agreements with Medtronic and Abbott. U.C. Nguyen has no competing interest.
